# Supplementary material for: High KYNU Expression Is Associated with Poor Prognosis, KEAP1/STK11 Mutations, and Immunosuppressive Metabolism in Patient-Derived but Not Murine Lung Adenocarcinomas
Source: Cancers (Basel). 2025 May 16;17(10):1681. doi: 10.3390/cancers17101681 (PMC12109616; doi:10.3390/cancers17101681)
Supplement: Supplementary file 1 [file cancers-17-01681-s001.zip › KYNU_supp_Figures_Apr2025.pdf]

## Figure S1

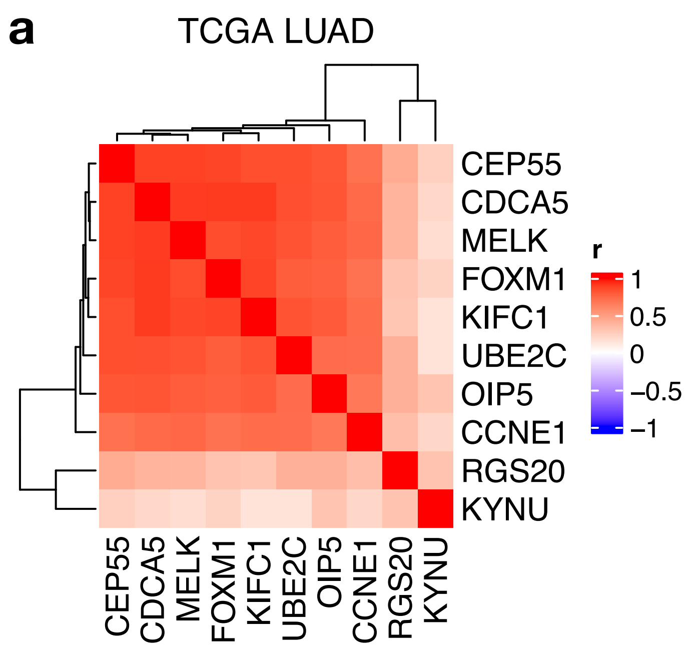


**Figure S1. *KYNU* Exhibits Weak Coexpression With Other Top Prognostic Genes**
**a-b.** Heatmaps showing Pearson correlation matrices for the top 10 prognostic genes identified using bimodal modeling, which outperforms median-based grouping in patient stratification (see Table S1). RNA-seq data used for the analysis are derived from TCGA LUAD **(a)** and CPTAC LUAD **(b)** cohorts**.** *KYNU* demonstrates weak coexpression with other prognostic genes in both datasets.

# Figure S2

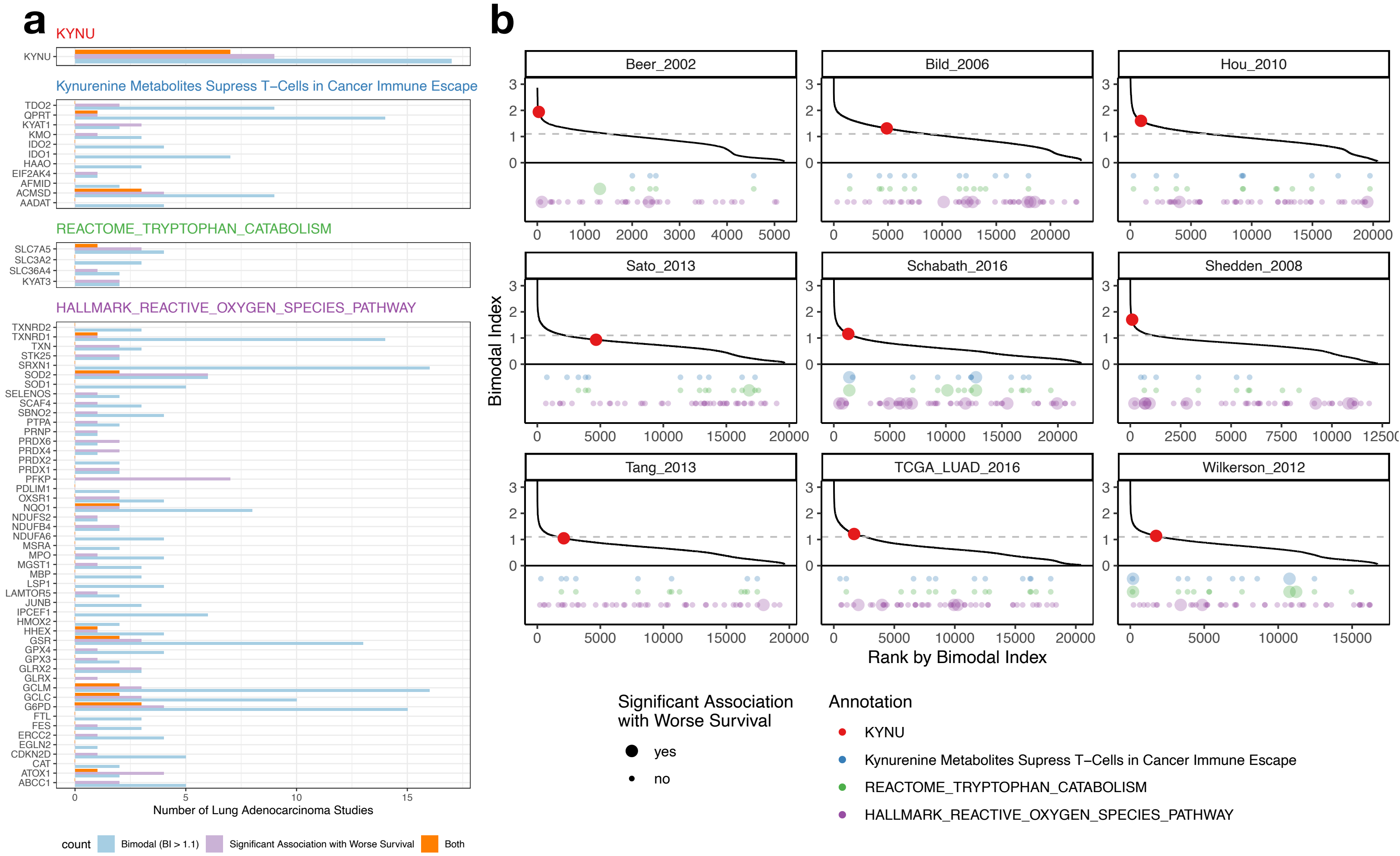


**Figure S2. Contextualization of *KYNU* within related pathways and its prognostic significance across LUAD studies**

**a.** Number of LUAD studies (n=23) in which *KYNU* or other genes from related pathways exhibited bimodal distribution (Bimodal Index > 1.1) (light blue bar), a significant association with worse overall survival ($p < 0.05$) (purple bar), or both (orange bar). Related pathways include "Kynurenine Metabolites Suppress T-Cells in Cancer Immune Escape," "Tryptophan Catabolism," and "Reactive Oxygen Species Pathway." *KYNU* was consistently identified as both bimodal and prognostically significant across the majority of studies. **b.** Rank position of *KYNU* and pathway-associated genes by descending bimodal index in the nine LUAD studies where *KYNU*-high clusters significantly correlated with worse overall survival. *KYNU* consistently ranked highest, underscoring its unique regulatory and prognostic role compared to other pathway members. Annotations highlight pathway groupings, and red points mark the rank of *KYNU* across studies.

# Figure S3

**a**

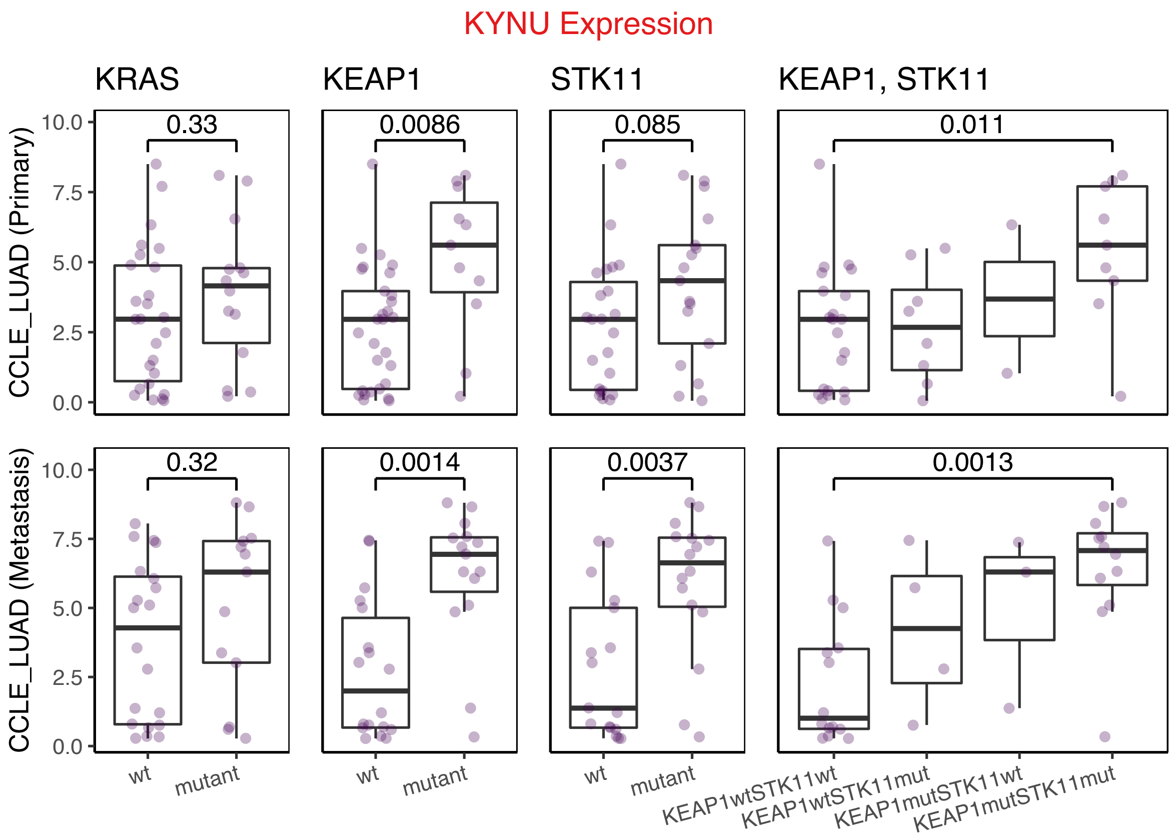


**b**

| | CCLE (primary) | CCLE (metastasis) | CCLE (combined) | TCGA | CPTAC |
|---|---|---|---|---|---|
| KEAP1wtSTK11wt | 52.5 | 42.4 | 49.3 | 70.1 | 71.8 |
| KEAP1wtSTK11mut | 20.0 | 12.1 | 16.0 | 11.3 | 13.6 |
| KEAP1mutSTK11wt | 5.0 | 9.1 | 6.7 | 11.9 | 8.2 |
| KEAP1mutSTK11mut | 22.5 | 36.4 | 28.0 | 6.7 | 6.4 |

**Figure S3. *KYNU* expression and mutation analysis in LUAD cell lines and tumor datasets**
**a**. *KYNU* expression stratified by *KRAS*, *KEAP1*, *STK11*, and *KEAP1/STK11* co-mutation status in LUAD cell lines derived from both primary and metastatic tumors (CCLE). P-values were calculated using the Wilcoxon rank-sum test. Elevated KYNU expression is most pronounced in cell lines harboring *KEAP1* and *STK11* co-mutations, particularly in metastasis-derived lines. **b**. Frequency distribution of KEAP1/STK11 oncogenotypes across CCLE (primary and metastatic cell lines), TCGA, and CPTAC LUAD datasets. KEAP1/STK11 co-mutations are more prevalent in CCLE datasets, potentially reflecting a selective advantages for in vitro establishment.

# Figure S4

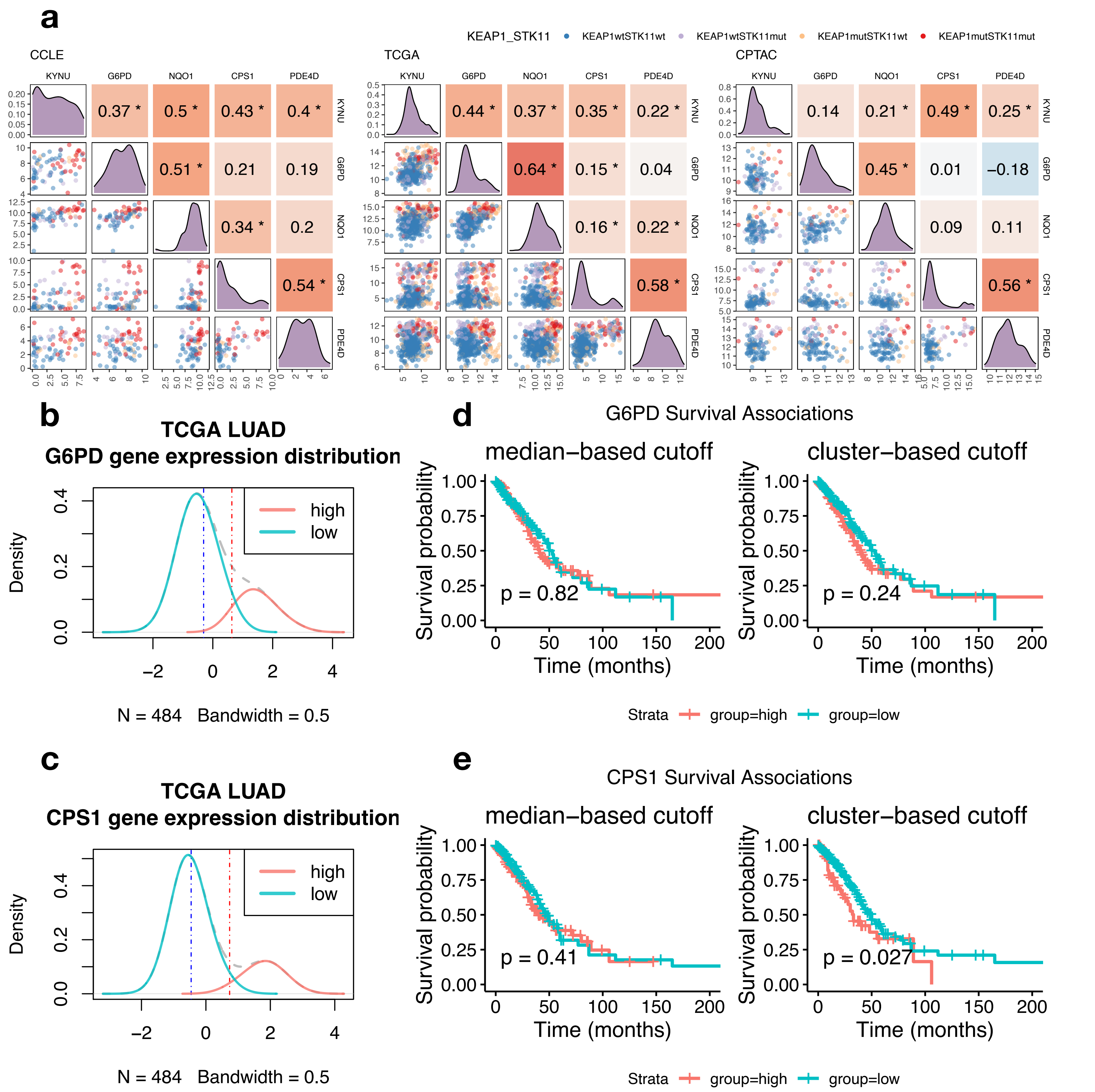


**Figure S4. Comparative Analysis of *KYNU* and other NRF2 and LKB1 Targets in LUAD**
**a.** Pairwise comparisons of *KYNU* with NRF2 targets (*G6PD* and *NQO1*) and LKB1 targets (*CPS1* and *PDE4D*) in CCLE, TCGA, and CPTAC LUAD datasets. Scatter plots (lower left panels), density plots (diagonal), and pairwise Pearson correlation coefficients (upper right panels) are shown. Asterisks (*) denote statistically significant correlations ($p < 0.05$)**. b-c**. Distribution of *G6PD* (**b**) and *CPS1* (**c**) expression in TCGA LUAD. Bimodal and median-based cutoffs for dichotomization into high- and low-expression groups are marked by red and blue dashed lines, respectively. **d-e.** Kaplan-Meier survival curves for TCGA LUAD patients stratified by *G6PD* (**d**) and CPS1 (**e**) expression using median-based (left) or model-based clustering (right) cutoffs. High expression is associated with distinct survival outcomes. Log-rank test p-values are shown.

# Figure S5

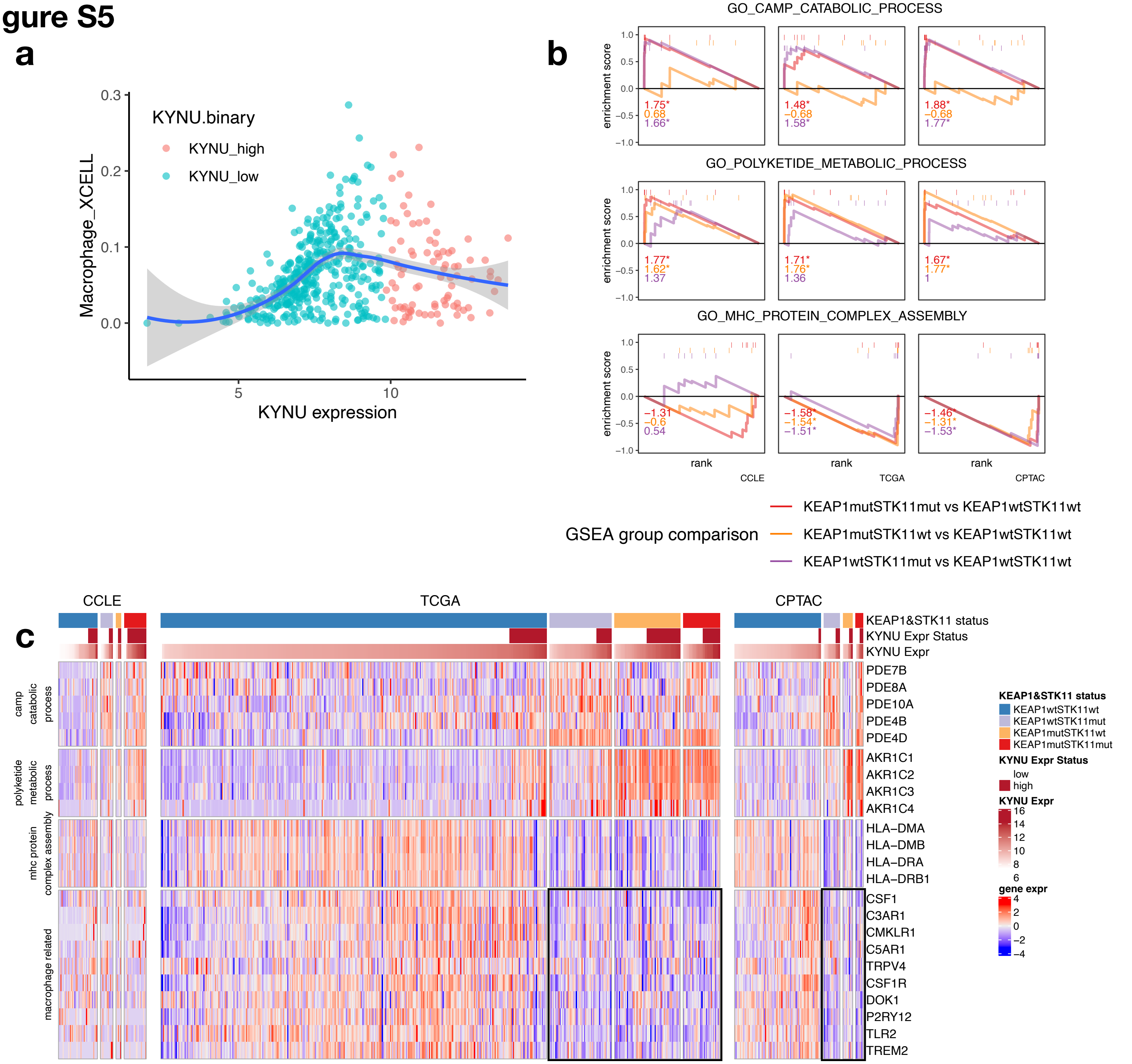


**Figure S5. Tumor Microenvironment and Gene Set Enrichment Analysis of KYNU-High Tumors**
**a**. In TCGA LUAD, KYNU-low tumors (blue) show a positive correlation with macrophage abundance, whereas KYNU-high tumors (red) do not. Dots represent individual tumor samples, and the curve represents a LOESS smoothed fit. **b**. Enrichment plots of selected pathways in LUAD samples from CCLE, TCGA, and CPTAC datasets. Group comparisons include double *KEAP1/STK11* co-mutants, single mutants, and wild-type samples. Normalized enrichment scores (NES) are displayed in the lower-left corner of each subplot, with an asterisk (*) indicating nominal p-values < 0.05. Highlighted pathways include cAMP catabolism, polyketide metabolism, and MHC II protein complex assembly. **c**. Heatmap showing the expression of selected genes from key pathways associated with *KEAP1/STK11* mutation status and *KYNU* expression status in LUAD samples. Data from CCLE, TCGA, and CPTAC datasets are included. Genes and pathways were selected based on their relevance to immune infiltration and cell-intrinsic metabolic processes. Black boxes highlight downregulation of macrophage-related genes in pateint tumors with single or double *KEAP1/STK11* mutations.

# Figure S6

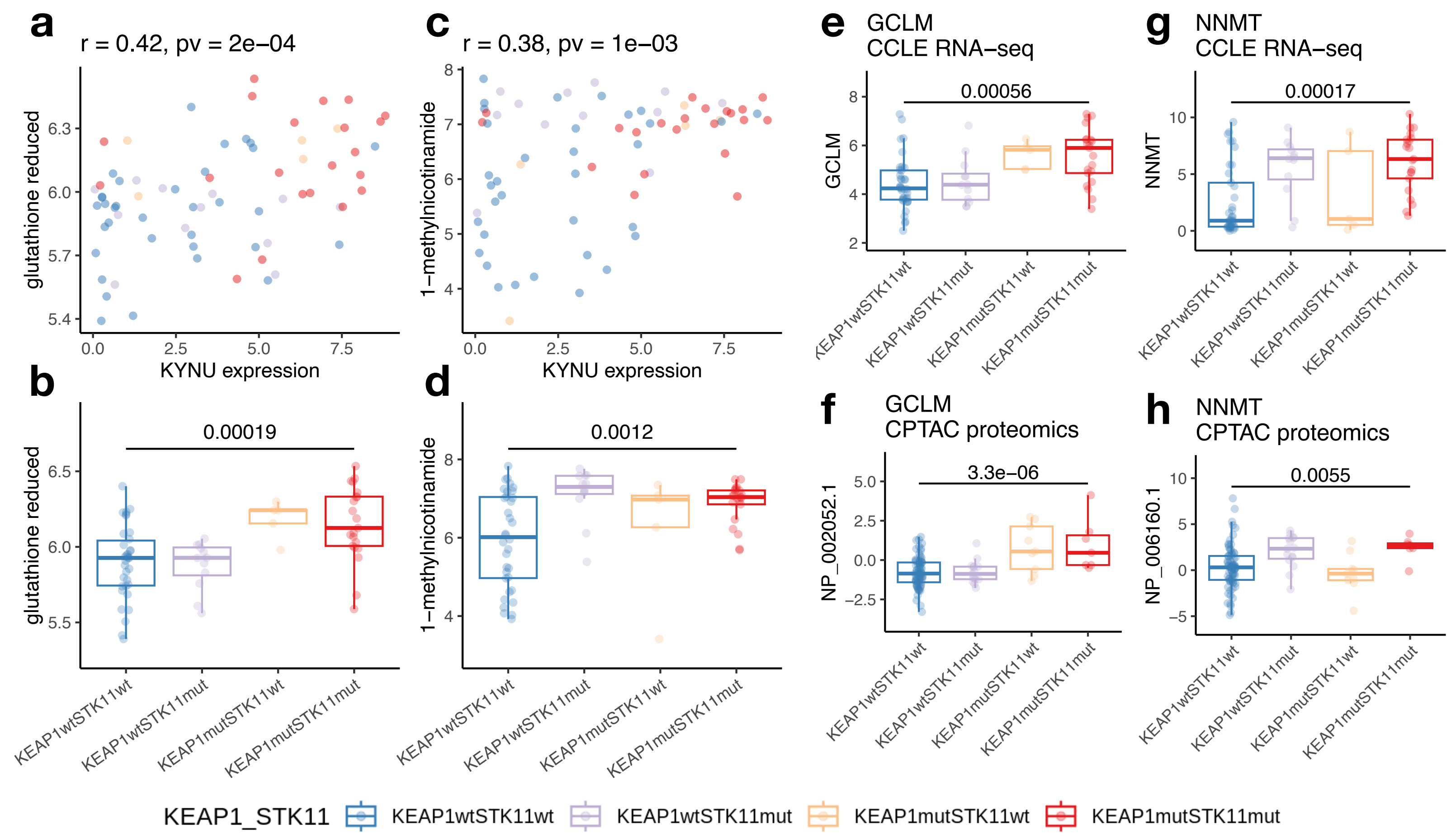


**Figure S6. KYNU Expression Correlates with Metabolic Reprogramming in KEAP1/STK11-Mutant LUAD**
**a**. Correlation between *KYNU* expression and reduced glutathione levels in LUAD cell lines. Pearson correlation coefficient (r) and p-value are shown. **b**. Reduced glutathione levels across *KEAP1*/*STK11* mutational groups. Higher glutathione levels are observed in KEAP1mut/STK11mut samples, consistent with NRF2-driven glutathione biosynthesis. **c**. Correlation between *KYNU* expression and 1-methylnicotinamide. **d**. 1-Methylnicotinamide levels across *KEAP1*/*STK11* mutational groups. **e**-**f**. GCLM mRNA expression in LUAD cell lines from CCLE RNA-seq data (**e**) and protein expression in LUAD tumors from CPTAC proteomics data (**f**). **g**-**h** *NNMT* mRNA expression in LUAD cell lines from CCLE RNA-seq data (**g**) and NNMT protein expression in LUAD tumors from CPTAC proteomics data (**h**). Box plots show expression levels across four groups: KEAP1wt/STK11wt, KEAP1wt/STK11mut, KEAP1mut/STK11wt, and KEAP1mut/STK11mut. P-values indicate significant differences in expression among the groups as assessed by one-way ANOVA. Increased expression of GCLM and NNMT in KEAP1mut/STK11mut samples supports the metabolic shifts observed in *KEAP1*/*STK11*-mutant tumors, consistent with NRF2-driven glutathione biosynthesis (GCLM) and LKB1-regulated nicotinamide metabolism (NNMT).
